# Genome-Wide Association Study for Weight Loss at the End of Dry-Curing of Hams Produced from Purebred Heavy Pigs

**DOI:** 10.3390/ani14131983

**Published:** 2024-07-05

**Authors:** Sara Faggion, Valentina Bonfatti, Paolo Carnier

**Affiliations:** Department of Comparative Biomedicine and Food Science, University of Padova, Viale dell’Università 16, 35020 Padova, Italy; sara.faggion@unipd.it (S.F.); paolo.carnier@unipd.it (P.C.)

**Keywords:** dry-cured ham, heavy pigs, protected designation of origin, GWAS, candidate genes, fat metabolism

## Abstract

**Simple Summary:**

Understanding the genetics of traits related to curing is crucial to both revealing the biological insights and effectively designing pig selective breeding programs. Ham weight loss after a prolonged dry-curing process was found to be polygenic, with multiple genomic regions across nearly all *Sus scrofa* chromosomes affecting the trait, each contributing a small amount of genetic variance. These regions were located in intergenic regions, regulatory regions (enhancer, promoter or open chromatin regions) and intronic regions of specific genes. Genes linked to curing-related traits (ham weight loss, drip loss, proteolysis), as well as genes specifically associated with animal growth and fat metabolism, were identified. The large number of genes associated with fat metabolism suggest that increasing the deposition of adipose tissue in pigs may be essential for ham curing aptitude.

**Abstract:**

Dissecting the genetics of production traits in livestock is of outmost importance, both to understand biological mechanisms underlying those traits and to facilitate the design of selection programs incorporating that information. For the pig industry, traits related to curing are key for protected designation of origin productions. In particular, appropriate ham weight loss after dry-curing ensures high quality of the final product and avoids economic losses. In this study, we analyzed data (N = 410) of ham weight loss after approximately 20 months of dry-curing. The animals used for ham production were purebred pigs belonging to a commercial line. A genome-wide association study (GWAS) of 29,844 SNP markers revealed the polygenic nature of the trait: 221 loci explaining a small percentage of the variance (0.3–1.65%) were identified on almost all *Sus scrofa* chromosomes. Post-GWAS analyses revealed 32 windows located within regulatory regions and 94 windows located in intronic regions of specific genes. In total, 30 candidate genes encoding receptors and enzymes associated with ham weight loss (*MTHFD1L, DUSP8*), proteolysis (*SPARCL1, MYH8*), drip loss (*TNNI2*), growth (*CDCA3, LSP1, CSMD1, AP2A2, TSPAN4*), and fat metabolism (*AGPAT4, IGF2R, PTDSS2, HRAS, TALDO1, BRSK2, TNNI2, SYT8, GTF2I, GTF2IRD1, LPCAT3, ATN1, GNB3, CMIP, SORCS2, CCSER1, SPP1*) were detected.

## 1. Introduction

The phenotypic variation of economically important complex traits in livestock can be described at different levels, including genomics, transcriptomics, and proteomics. Multi-omics approaches are used to identify molecular markers and elucidate their role in shaping phenotypes along with their underlying functional and regulatory mechanisms [1]. Over the past decades, genome-wide association studies (GWAS) have identified several gene loci (quantitative trait loci, QTL) responsible for variations in quantitative economic traits in livestock [2,3,4,5]. Recently, GWAS have been coupled with post-GWAS analyses aimed at locating genetic variants and detecting the possible functional mechanisms that affect traits under selection [1,6].

Within the pig breeding industry, significant efforts have been directed to dissecting the genetics of production traits [7,8,9] and those related to curing [3,10,11,12], mostly driven by the economic importance of the dry-cured ham market in Europe [13,14].

In Italy, dry-cured ham marked with the European “protected designation of origin” (PDO) label represents the most valuable product in the pig breeding industry [15], with almost 75% of slaughtered pigs used for PDO productions [16]. The PDO production process relies solely on salt as a preservative, and on the control of temperature and humidity to achieve optimal dryness and ham sensorial properties. Dehydration is a key process during dry-curing, but excessive ham weight loss is undesired as it impairs the quality of the final product leading, in turn, to a decrease in marketable product and economic losses. As the quality of the raw thighs is a critical aspect affecting ham weight losses during dry-curing, since the early 1990s, pig breeding companies providing pigs intended for PDO productions have focused on improving the quality of raw hams with the aim of decreasing ham weight loss and using ham weight loss at first salting (WLS) as an indicator of ham weight loss at the end of curing (WLC). Genome-wide association studies performed on WLS in Italian Duroc and Large White pig breeds have demonstrated the polygenicity of the trait and revealed several QTL regions significantly associated with WLS which were mapped on all *Sus scrofa* chromosomes (SSCs) except SSC16 and SSC18 [10,12]. More than 70 genes within the detected windows were also identified [12]. 

So far, studies have focused only on WLS, as the phenotypes can be more easily and quickly recorded compared to phenotypes of weight loss at the end of a prolonged curing period, as PDO productions required. However, since the estimated genetic correlation between WLS and WLC is 0.65 [17], a significant proportion of genetic variation in WLC is not explained by WLS. Hence, it is worthwhile investigating the associations between WLC and genomic regions in pig populations where breeding goals focus on ham quality. Moreover, detecting QTLs potentially associated with WLC and dissecting this trait by identifying candidate genes could contribute to a better understanding of functions and biological mechanisms affecting curing aptitude in heavy pigs. This could also facilitate the incorporation of that information into the design of customized selection programs [18], aiding the transition from conventional breeding to molecular breeding, exploiting not only one level of genomic information (such as marker-assisted selection or genomic selection) but incorporating data from multi-omics studies that could help explain the molecular basis of phenotypic differences in complex traits [1].

In this study, we performed a genome-wide association study for WLC at the end of a dry-curing process that lasted approximately 20 months. The animals used in the study were purebred heavy pigs belonging to a commercial line specifically selected for dry-cured ham quality traits. Post-GWAS analyses included SNP location, candidate gene search, and gene network analyses to detect possible functional mechanisms associated with WLC.

## 2. Materials and Methods

### 2.1. Animals and Experimental Trial

The data used in this study were collected during an experiment aiming to investigate how different pig rearing systems and diets affect performance, carcass, and dry-cured ham quality. The dataset used in this study included a total of 420 purebred C21 Goland (Gorzagri, Fonzaso, Italy) pigs (205 gilts and 215 barrows) offspring of 23 sires and 128 dams. The C21 Goland pig line is selected for traits related to the quality of raw hams and thighs suitability for dry-curing. Experimental pigs were raised on a single farm and fed identical commercial diets until they were transferred, in 4 subsequent batches (N = 97 to 111 pigs), to the experimental station of the University of Padova (Legnaro, Italy). The average weight and age of the animals at the arrival at the experimental station was 93.6 ± 8.8 kg and 148 ± 1 days old, respectively. The experiment was performed from June 2018 to July 2020. Pigs from each batch were housed in 8 pens randomly assigned to two feeding groups: ad libitum (N = 207) and restricted feeding (N = 213). Each pen was 5.8 × 3.8 m in size and the rearing density was 1.57 m^2^/pig, corresponding to 12 to 15 pigs for each pen. A single-space electronic feeder (Compident Pig–MLP, Schauer Agrotronic, Prambachkirchen, Austria) was installed in each pen to ensure that each pig received the recommended daily amount of food. Each pen had nipple drinkers with unrestricted access to water. Within each feeding group, pens were assigned to two treatments (2 pens per treatment). For the ad libitum-fed group, the 2 treatments were both characterized by a high-protein diet with no limitations on indispensable amino-acid content, but the target was slaughter weight at 170 kg (HP170) or age at slaughter equal to 270 days (HP9M). For the restricted-fed group, the treatments consisted of a medium-protein (MP) or a low-protein (LP) diet; pigs subjected to the MP treatment were slaughtered at 270 days of age and 170 kg BW (traditional Italian heavy pig farming system), whereas pigs subjected to the LP treatment had the target of weight at slaughter equal to 170 kg BW but variable age at slaughter (from 251 to 289 days). The ingredients and the nutrient content of the diets are described in detail in Malgwi et al. [19] and in Schiavon et al. [20]. The pigs were fed diets designed to meet the nutrient requirements for pigs of their size, either fully or partially [20,21].

### 2.2. Measurement of Ham Weight Loss at the End of Dry-Curing

Pigs were slaughtered according to standard commercial slaughter procedures [22] at the same slaughterhouse (OPAS, Carpi, Italy), on different dates according to their batch and rearing strategy. Hot carcasses were sectioned to obtain the hams. After 24-h cooling (1–2 °C), green hams were trimmed to obtain the characteristic round shape and, within two days after slaughter, left thighs were transferred to the ham factory (“Attilio Fontana prosciutti”, Montagnana, Padova, Italy) to be processed according to the product specification of the “Prosciutto Veneto Berico-Euganeo” [23]. The entire ham dry-curing process was described in detail by Toscano et al. [24]; briefly, hams were subjected to the following steps: (i) first salting (6 days at a temperature of 2–3 °C and 75 to 95% humidity); (ii) second salting (5 to 8 days depending on ham weight, at 2–3 °C and 75 to 95% humidity); (iii) desalting using compressed air and pressing to complete ham bleeding and salt absorption; (iv) pre-resting (14 days at 1–3 °C); (v) resting (90 days at 2–4 °C and 70 to 80% humidity); (vi) washing with warm water (40 °C); (vii) drying (overnight at 20 °C and 90% humidity); (viii) pre-curing (40 days at 12–16 °C); (ix) “grouting” (ham covering with a mixture of rice flour and lard), (x) curing (approximately 450 days). At the end of the entire dry-curing process (approximately 20 months), the weight of each dry-cured ham was recorded and WLC was then computed as the difference between the weight of the trimmed green ham and the weight of the dry-cured ham and expressed as a percentage of the initial weight.

### 2.3. Genotyping

DNA extraction and genotyping were performed at the GeneSeek Inc. laboratory (Lincoln, NE, USA) from muscular tissue samples collected from each of the 420 experimental pigs. Animals were genotyped using a high-density SNP chip (GGP Porcine 50K). In total, 410 animals out of 420 were successfully genotyped. SNP markers were quality filtered using an in-house script in R software v. 4.2.3 [25]; SNPs with a minor allele frequency (MAF) lower than 1% and call rate lower than 95% were discarded. The FImpute software v. 2.2 [26] was used to impute missing genotypes exploiting genotype data and pedigree information available for the C21 Goland pig line. The final number of available SNP genotypes per animal was 29,844.

### 2.4. Genetic Parameters and Genome-Wide Association Study for Ham Weight Loss at the End of Dry-Curing

Variance components and heritability of WLC were estimated using the average information restricted maximum likelihood method implemented in the BLUPf90+ program suite [27] with the following univariate mixed linear model:

**y** = **Xb** + **Uc** + **Wa** + **e** (1)

where **y** is the vector of phenotypes (WLC), **b** is the vector of the fixed effects of sex (2 levels) and treatment (4 levels), **X** is the incidence matrix relating **y** to **b**, **c** is the vector of the random effect of the batch (4 levels; batch effect included the effect of season and slaughter date), **U** is the incidence matrix relating **y** to **c**, **a** is the vector of random animal additive genetic effects, **W** is the incidence matrix relating **y** to **a**, **e** is the vector of the random residuals assumed to be N(0, I$\sigma_{e}^{2}$), with N(·) indicating a normal probability density function, **I** being an identity matrix of appropriate order, and $\sigma_{e}^{2}$ being the residual variance. The vector **a** of random animal additive genetic effects was assumed to be distributed as N(0, A$\sigma_{a}^{2}$), where **A** is the numerator relationship matrix between animals (from pedigree data of 1917 animals spanning 22 generations, 410 animals with phenotype and 1507 ancestors including the founder animals of the C21 Goland line) and $\sigma_{a}^{2}$ is the additive genetic variance.

A GWAS was performed to test the association between WLC and SNP genotypes. The analyses were performed following a genomic BLUP (GBLUP)-GWAS procedure using BLUPf90+ program [27]. Firstly, genomic-based estimated breeding values (GEBVs) were predicted using model (1), replacing the A matrix with the genomic relationship matrix (from genomic data, G matrix).

G was obtained following the method of VanRaden [28]:

(2) $$G=\frac{ZZ\prime }{2\sum_{i=1}^{m}p_{i}{(1-p}_{i})}$$

where **Z** is a matrix of gene content (0, 1, 2) adjusted for allele frequencies; *m* is the number of SNPs, and *p_i_* is the minor allele frequency of the *i*-th SNP.

The allele substitution effect of each SNP was derived from GEBV using postGSf90 [29]:

(3) $$\hat{g}=\frac{1}{2\sum_{i=1}^{m}p_{i}{(1-p}_{i})}Z\prime G^{-1}\hat{u}$$

where $\hat{g}$ is the vector of SNP effects and $\hat{u}$ is the vector of GEBV. 

SNP effects derived from GEBV were used to compute the SNP variance explained. The explained percentage of genetic variance was calculated using moving windows of adjacent SNPs spanning a distance of 0.4 Mb, which corresponds to the average haplotype block size observed in commercial pig lines [30]. 

*p*-values were obtained as described in Aguilar et al. [31]:

(4) $${p-value}_{i}=2(1-\Phi \left(\left|\frac{{\hat{g}}_{i}}{sd\left({\hat{g}}_{i}\right)}\right|\right))$$

where Φ is the cumulative standard normal distribution and sd(**ĝ_i_**) is the square root of the variance of the *i*-th SNP effect estimate, obtained as in Gualdrón-Duarte et al. [32]. 

The probability of committing a type I error (i.e., rejecting the null hypothesis of no association when the hypothesis is true) was set to 0.00005 (genome-wide significance threshold) and 0.0005 (suggestive significance threshold), as previously defined by several studies on livestock [7,10,12,33] and the Wellcome Trust Case Control Consortium [34]. 

The inflation of the *p*-values was estimated as the median of the resulting χ^2^ test statistics divided by the expected median of the χ^2^ distribution (λ factor). The R package *qqman* [35] was used to obtain the quantile–quantile (Q-Q) plot of the observed *p*-values for each SNP against identically uniformly distributed *p*-values, the Manhattan plot of GBLUP-GWAS results, and the plot of the variance explained by SNP moving windows.

### 2.5. Candidate Gene Search for Ham Weight Loss at the End of Dry-Curing and Gene Network Analysis

The 0.4 Mb windows explaining 0.3% or more of the total genetic variance were selected for subsequent candidate gene search analyses. The threshold of 0.3% was based on the expected contribution of SNP windows [36]. Assuming that all the windows collectively explain 100% of the genetic variance, and each window contributes equally, the expected proportion of variance explained by each window would be 1 divided the total number of windows obtained in the GWAS analysis. As moving windows were used, 29,702 window segments were obtained, thus each window would theoretically explain 0.003% of the genetic variance. The threshold of 0.3% used in this study was 100 times higher than the expected variance explained (0.003% × 100 = 0.3%). Based on the starting and ending coordinates of each window, functional annotations and genetic locations of SNPs were obtained through the Ensembl VEP (variant effect predictor) tool (https://www.ensembl.org/info/docs/tools/vep/index.html; accessed on 16 April 2024). The candidate genes located within 200 kb upstream and downstream the significant SNPs were identified by the Biomart platform on Ensemble (https://www.ensembl.org/biomart/martview/; accessed on 17 April 2024) using the gene annotation information of the *Sus scrofa* reference genome (Sscrofa11.1).

A gene network analysis was performed using the plug-in GeneMania [37] of the Cytoscape software v. 3.10.2 [38] to find potential interactions (protein and genetic interactions, shared pathways, co-expression, co-localization, and protein domain similarity) between the identified genes.

## 3. Results and Discussion

### 3.1. Descriptive Statistics and Heritability for Ham Weight Loss at the End of Dry-Curing

For the animals included in the study (N = 410), the average weight at slaughter (±SD) was equal to 176.30 ± 14.76 kg and ranged from 137.60 to 225.10 kg. The entire ham dry-curing process lasted on average 604 days and ranged from 566 to 631 days. The mean (±SD) of ham weight loss during dry-curing (%) was 28.95 ± 2.18%; the values ranged between 22.98 and 36.22%. 

The percentage of WLC was consistent with reports on Italian dry-cured hams (~30% of the initial weight) [39] and, as expected, higher than those recorded after 12 months of dry-curing on crossbreds generated by the nucleus boars of the C21 Goland sire line (27.8%) [40,41,42]. 

Estimates of additive genetic standard deviation ($\sigma_{a}$) and residual standard deviation ($\sigma_{e}$) for WLC (%) were 1.14% and 1.62%, respectively. The estimated heritability was moderate (h^2^ ± SE: 0.33 ± 0.01), but in the range of h^2^ estimates obtained for crossbred pigs using more than 1600 phenotypes of WLC after 12 months of dry-curing (0.27–0.31) [40,41,42] and h^2^ estimates reported in the literature for WLS (0.30 to 0.61) [17,43].

### 3.2. Genome-Wide Association Study for Ham Weight Loss at the End of Dry-Curing

**Figure 1:**
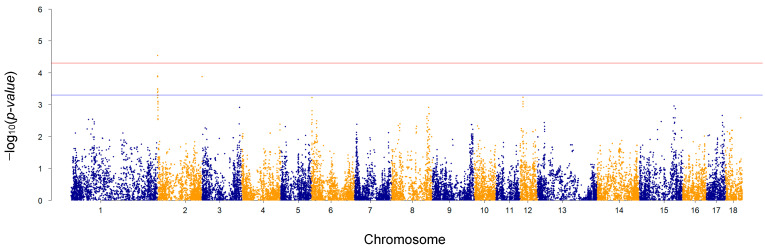
Genome-wide association plot for ham weight loss at the end of dry-curing. The red horizontal line identifies the genome-wide significance threshold (probability of committing a type I error equal to 0.00005) and the blue horizontal line identifies the suggestive significance threshold (probability of committing a type I error equal to 0.0005).

Genome-wide and suggestive significance thresholds after −log_10_ transformation were computed to be 4.3 and 3.3, respectively. The GWAS identified only one SNP beyond the genome-wide significance threshold and 15 SNPs beyond the suggestive significance threshold, all located on SSC2 (Figure 1). 

The capacity of GWAS to pinpoint genomic regions significantly associated to trait variation is strictly related to sample size and phenotypic variability, which in turn affect the statistical power of the test. Despite the restricted number of samples available for our study due to the operational challenges of the experimental procedures, associations between SNP markers and WLC were detected. It is worth mentioning that the phenotypic variation within our dataset is consistent to those observed for large datasets including more than 1600 phenotypes [40,41,42]. However, the current experimental trial involved animals fed ad libitum a high protein diet and animals fed restricted either a low or medium protein diet, resulting in ad libitum-fed animals with increased fat thickness and lower ham WLC compared to hams of restricted-fed animals (Mondin et al., pers. comm.).

**Figure 2:**
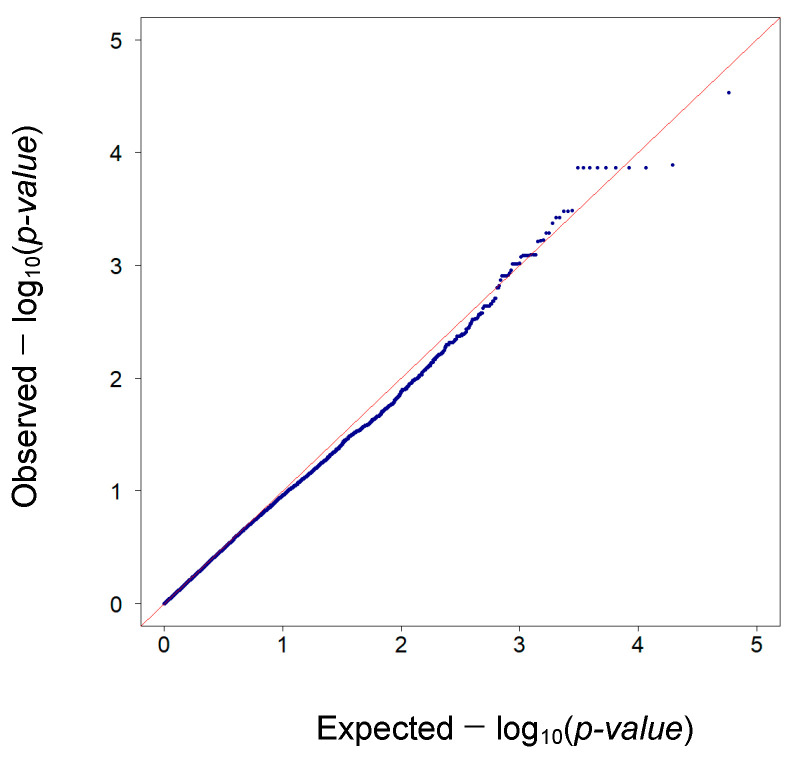
Q-Q plot representing the observed *p*-values for each SNP plotted against identically uniformly distributed values, which would be expected under the null hypothesis of no association. The diagonal represents the expected distribution of the −log_10_(*p*-values), blue points represent the observed distribution of the −log_10_ (*p*-values).

The Q-Q plot of the observed *p*-values for each SNP (Figure 2), which represents graphically the deviation of quantiles for the observed *p*-values from those expected under the null hypothesis, confirmed the results observed for the GWAS; in fact, the tail of the distribution deviated from the null hypothesis, suggesting that several SNP are likely to be associated with WLC. In the Q-Q plot, the observed *p*-values for each SNP are sorted from the highest to the lowest, and plotted against identically uniformly distributed *p*-values, which would be expected under the null hypothesis of no association. On the plot, observed and expected values are −log_10_-transformed *p*-values. Deviations from the diagonal suggest deflation or inflation in *p*-values; in particular, when points lie below the diagonal, observed *p*-values are smaller than expected under the null hypothesis and association might be hypothesized, whereas an early separation of the expected from the observed *p*-values, with points lying above the diagonal, often suggests population stratification [44]. In our case, the Q-Q plot did not show significant deviations from the null hypothesis, indicating that GBLUP accurately captured the population structure through the relationship matrices. The lambda (λ) factor, which is normally used as an indicator of the inflation of the *p*-values, was equal to 0.91. The inflation factor λ is defined as the median of the resulting χ^2^ test statistics divided by the theoretical median under the null distribution [45]. Assuming that the median of a χ^2^ distribution with one degree of freedom is 0.45 and the results follow the χ^2^ distribution, the expected λ value is equal to 1, and no *p*-value inflation is hypothesized. If the value of λ is equal to or lower than 1, no adjustments for population stratification are necessary and data can be tested with an acceptable risk of false positives [46,47].

**Figure 3:**
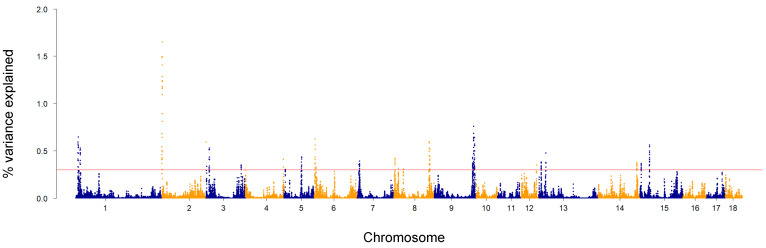
Percentage of genetic variance explained by SNP windows for ham weight loss at the end of dry-curing. Each dot represents one SNP window of 0.4 Mb. The horizontal line identifies the threshold of 0.3% used to select SNPs for candidate gene search.

To select SNP markers potentially associated with complex traits, several authors [48,49,50] have proposed approaches based on the percentage of variance explained. Following this strategy, we have detected signals from several chromosome regions, most of them consistent with studies on WLS [10,12]. The percentage of variance explained by each SNP window for WLC is depicted in Figure 3. We detected 221 windows explaining more than 0.3% of the genetic variance, and up to 1.65% of the genetic variance. These regions were detected on SSC1 (25 windows), 2 (30 windows), 3 (19 windows), 4 (2 windows), 5 (10 windows), 6 (11 windows), 7 (8 windows), 8 (22 windows), 9 (51 windows), 12 (1 windows), 13 (13 windows), 14 (9 windows), and 15 (20 windows). The most relevant windows were located on SSC2 and included the same SNPs identified as significantly associated to WLC (Figure 2). Details on the exact position, −log_10_-transformed *p*-value, and percentage of variance explained by each SNP included in the 221 windows are reported in Table S1. The detection of multiple genomic windows, each explaining a small amount of WLC additive genetic variance, supports the hypothesis of the polygenic nature of the trait, which has been already suggested by GWAS targeting WLS. In those studies, several genetic markers significantly associated with trait variation were identified: Fontanesi et al. [10] reported 29 genomic regions in a population consisting of more than 1300 Italian Duroc pigs (on SSC1, 2, 3, 4, 5, 7, 8, 9, 10, 11, 13, 14, 15, 17), whereas, in 574 Large White pigs, seven regions located on SSC2, 4, 6, 7, 8, and 12, were detected by Bertolini et al. [12]. In Fontanesi et al. [10], SSC16 and SSC18 were the only chromosomes that did not include genomic regions significantly associated with WLS. Similarly, in our study, SSC16 and SSC18 were among the chromosomes not detected as associated with WLC. The similarities might be partly explained by the genetic correlation between WLS and WLC (0.65) [17]. Moreover, even if WLS and WLC are correlated traits and underlying mechanisms might be similar, some functions and biological mechanisms might vary as the curing process progresses.

### 3.3. Genetic Marker Locations, Candidate Genes and Gene Network Analysis

**Figure 4:**
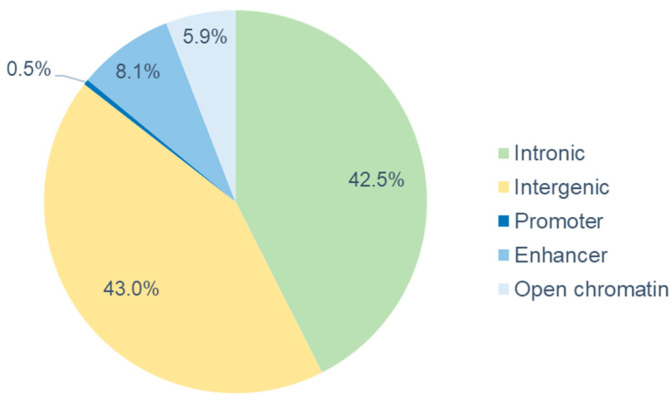
Percentage of SNPs located in enhancer, promoter, open chromatin, intronic, and intergenic regions.

Out of 221 windows explaining more than 0.3% of the genetic variance, 32 (14.5%) were located in regulatory regions (enhancer, promoter or open chromatin regions, 94 (42.5%) were located in intronic regions of specific genes, and 95 (43.0%) were intergenic variants (Figure 4). The 32 windows in regulatory regions were identified on SSC1, 2, 3, 5, 6, 7, 8, and 13 (details are reported in Table S2). The majority of the windows were positioned in enhancer regions (8.1%), whereas 5.9% and 0.5% out of the total were located in open chromatin and promoter regions, respectively. Open or accessible regions of the genome are considered the most important locations for regulatory elements [51], as the accessibility of chromatin affects gene expression in tissue cells, playing a crucial role in regulation. In particular, promoters and enhancers are regulatory elements that facilitate the transcription of nearby genes [52]. Promoters start the transcription at self-contained transcription start sites [53], whereas enhancers are cis-regulatory sequences that activate transcription initiation at promoters [54] and interact with transcription factors to modulate gene expression [55]. 

**Figure 5:**
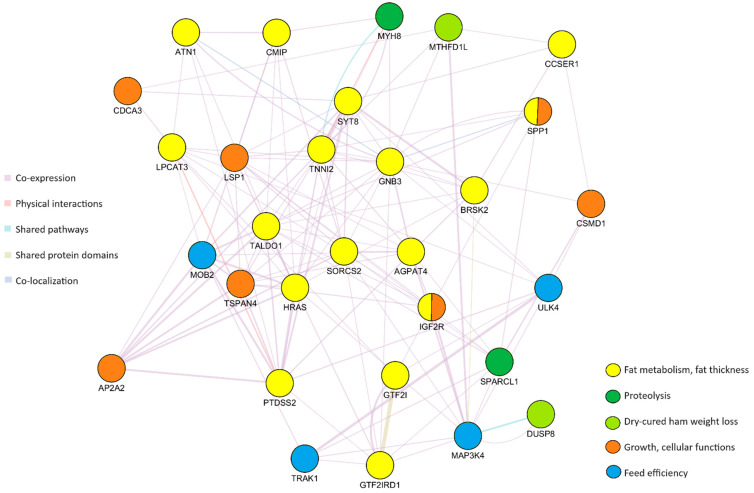
Gene network for ham weight loss at the end of dry-curing. Genes are characterized by colors according to their functions in pigs (fat metabolism or fat thickness, proteolysis, dry-cured ham weight loss, growth or cellular functions, feed efficiency), whereas different colors of the lines represent the different interconnections between genes due to co-expression, physical interactions, shared pathways, shared protein domains or co-localization.

Introns are important elements that reside within a gene and that play several roles, including gene expression regulation and enhancement [56]. Among the 94 windows located within intronic regions of genes, we identified 30 genes encoding receptors or enzymes associated with fat metabolism, backfat thickness, growth performances, carcass traits and feed efficiency in pigs, proteolysis, or dry-cured ham weight loss at first salting (Table 1). The gene network analysis results are depicted in Figure 5. A dense co-expression network among the identified candidate genes (93.87%) was observed. Few genes were physically interconnected (2.08%), shared pathways (1.65%) or protein domains (1.35%), or were co-localized (1.05%).

Among the identified candidate genes, *MTHFD1L* (*methylenetetrahydrofolate dehydrogenase NADP+ dependent 1-like*) and *DUSP8* (*dual specificity phosphatase 8*) have been previously associated with WLS in Italian Large White pigs [11]. *MTHFD1L* is associated with the synthesis of tetrahydrofolate in the mitochondrion and its related pathways are metabolism of water-soluble vitamins. Besides being associated with WLS, it has been associated with the number of ribs and carcass length in Suhuai pigs [57]. The *DUSP8* gene encodes a member of the dual-specificity protein phosphatase (DUSP) family and is involved in regulating signaling pathways that are critical for cellular processes such as growth, proliferation, differentiation, and stress responses. Some members of the DUSP family are known to regulate the mitogen-activated protein kinase (MAPK) signaling pathway, which is involved in cell growth and differentiation, and this pattern is confirmed by our network analysis results where *DUSP8* and *MAP3K4* (*mitogen-activated protein kinase 4*) were identified as sharing pathway genes (Figure 5).

*SPARCL1* (*sparc-like 1*) belongs to the SPARC family of extracellular glycoproteins. It is involved in cellular metabolism and regulates calcium ions binding activity [58]. Similarly, analyses of the transcriptome of *longissimus dorsi* muscle in Iberian pigs identified the *MYH8* (*myosin heavy chain 8*) gene as involved in calcium signaling pathways [59]. The regulation of calcium release into the cytoplasm is a biological process related to proteolysis, which is key during dry-curing. Proteolysis (i.e., degradation of meat proteins by endogenous proteolytic enzymes) occurring in ham muscles leads to increased concentrations of smaller peptides and free amino acids [60]. The activity of proteolytic enzymes dictates the extent of proteolysis affecting the texture of dry-cured ham by degrading large cytoskeletal and myofibrillar proteins, which in turn has an impact on meat tenderness [61]. Post-mortem proteolysis, particularly the degradation of cytoskeletal proteins like desmin, has been reported to affect drip loss [62,63]. Additionally, the degradation of integrin, which connects the cell membrane to the cytoskeleton, may be correlated to the formation of drip channels in pork [64].

The large majority of the identified genes were associated with lipid metabolic processes and to variations in fat thickness and intramuscular fat. These genes are strictly related in the network, as almost all of them are co-expressed (Figure 5). In general, subcutaneous fat is crucial during the curing process, as it protects the muscular fraction from the dehydrating effect of salt by limiting exchanges between the muscle and the external environment, leading to reduced ham weight loss during curing [39,65], whereas leaner hams tend to have higher salt content due to increased weight loss [61]. For this reason, breeding programs targeting pigs intended for PDO productions aim to maintain or enhance adipose tissue deposition [66].

*AGPAT4* (*1-acylglycerol-3-phosphate O-acyltransferases 4*) catalyzes the conversion of lysophosphatidic acid to phosphatidic acid, which is a precursor of triacylglycerol; in Duroc pigs, significant associations between this gene and intramuscular fat content and fatty acid composition have been reported [67]. *IGF2R* (*insulin-like growth factor receptor*) has been reported in association with pig carcass traits and quality; it has major effects on muscle growth and its functions can be related to fat metabolism and backfat thickness in different pig genetic lines [68,69]. *PTDSS2* (*phosphatidylserine synthase 2*), *HRAS* (*GTPase HRas proto-oncogene*), and *BRSK2* (*BR serine/threonine kinase 2*) have been associated with fat deposition and backfat thickness in pigs [68]. Bergamaschi et al. [9] have also reported *PTDSS2* and *HRAS* as associated to loin depth in the Duroc breed. *LPCAT3* (*lysophosphatidylcholine acyltransferase 3*) gene expression affects lipid accumulation in pigs, regulating intramuscular and subcutaneous adipocyte biogenesis [70]. *PTDSS2*, *LPCAT3*, and *TSPAN4* (*tetraspanin 4*) have been found to be physically interconnected due to physical contact between gene products (i.e., proteins), that can influence gene functions and biological processes. If the interaction between *PTSSD2* and *LPCAT3* is predictable, due to the association of both genes with fat deposition, the relationship with *TSPAN4* can be only hypothesized. *TSPAN4* affects the regulation of cell development, activation, growth, and motility encoding cell-surface proteins [71], thus we can speculate that it might be involved with adipocyte biogenesis due to the affinity with *PTSSD2* and *LPCAT3*. *TALDO1* (*transaldolase 1*) is a key enzyme involved in the nonoxidative pentose phosphate pathway and provides NADPH for lipid biosynthesis [72]. The *Troponin I2* (*TNNI2*) gene has been associated with fat percentage, lean meat percentage, loin eye area, thorax–waist backfat thickness, and average backfat thickness in pigs [73]; *TNNI2* has been reported to affect significantly muscle pH, color, marbling, intramuscular fat content [74], and drip loss [75]. In our network analysis, *TNNI2* was observed to share the pathway with *MYH8*, meaning that the genes contribute to or regulate the same functions or outcomes, often working together to perform complex tasks (i.e., metabolism, cell signaling, or gene expression regulation). In particular, as *TNNI2* was associated with fat content and *MYH8* was associated with proteolysis, both key processes during dry-curing, these genes might be linked to curing aptitudes. *Synaptotagmin 8* (*SYT8*) has been associated with lipid metabolism and insulin secretion; it encodes a member of the synaptotagmin protein family, which consists of membrane proteins involved in neurotransmission and hormone secretion. The Ca^2+^ non-binding isoform, syt 8, has been found in insulin-secreting cells. Studies have shown that glucose stimulates *SYT8* expression in human islets, and knocking down *SYT8* impairs insulin release [76], although the precise role of syt 8 in insulin secretion is still unclear [77]. *GTF2IRD1* (*GTF2I repeat domain-containing 1*) and *GTF2I* (*general transcription factor IIi*) are two genes sharing protein domains and were found to be expressed mainly in human brown fat adipocytes [78,79]. In Duroc pigs, they have been associated with intramuscular fat content [80]. The expression of *CMIP* (*C-Maf inducing protein*) was described as involved in lipid metabolism in the pig skeletal muscle [81]. *SORCS2* (*Sortilin-Related VPS10 Domain-Containing Receptor 2*) belongs to the same family of *SORCS1* and *SORCS3*, two related sorting receptors expressed in neurons of the arcuate nucleus of the hypothalamus and related to fat deposition in mice [82]. *SORCS1* and *SORCS3* were recently associated with backfat traits and fat deposition in Duroc pigs [83], whereas Júnior et al. [84] identified the *SORCS2* gene as significantly related to backfat traits of Nellore cattle. *Coiled-Coil Serine-Rich Protein 1* (*CCSER1*) was defined as involved in mechanisms of fat deposition, in particular backfat thickness in Chuying-black pigs [85]. *ATN1* (*atrophin 1*), *GNB3* (*G protein subunit beta 3 or guanine nucleotide-binding protein*), and *SPP1* (*secreted phosphoprotein 1*) have been found to be located in a region previously associated with fat deposition and lipolysis. In the study by Serão et al. [86], the expression pattern of *ATN1* indicated biochemical pathways related to fat deposition in the early stages of development of pigs to prevent adipose hypertrophy. *GNB3* has been reported to be involved in energy homeostasis and promotion of lipolysis; in pigs, it was located within or near QTL for fatness traits, in particular backfat thickness [87]. The *SPP1* gene is involved in bone morphogenesis [88]. It has been associated with growth and carcass traits in a crossbred population derived from Landrace and Korean Black pigs; in particular, polymorphisms of the *SPP1* gene have demonstrated to have effects on growth rate, body length, backfat thickness, and loin muscle area [89].

Several genes were associated with growth or cellular function regulation. *CDCA3* (*cell division cycle associated 3*) was described as a candidate gene associated with body weight in Yorkshire pigs [90]. *LSP1* (*lymphocyte-specific protein 1*) has been reported to stimulate specific myogenic factors in Bísaro pig breed, thus affecting skeletal muscle development [91]. *CSMD1* (*CUB and sushi multiple domains 1*) has been shown to control cellular functions, affecting intracellular signaling and interacting with growth factor receptors [92]; this suggests a potential association between the *CSMD1* gene and growth in pigs. 

*AP2A2* (*adaptor-related protein complex 2 subunit alpha 2*) encodes for a protein (a subunit of the AP-2 adaptor protein complex) which is involved in linking lipid and protein membrane components with the clathrin lattice. This interaction supports the formation of clathrin-coated vesicles, and the encoded subunit aids in the process by binding lipids in the cell membrane. In a functional analysis performed on Korean native pig and Yorkshire breeds, *AP2A2* was involved in epidermal growth factor signaling [93]. 

Genes associated with feed efficiency traits were *ULK4* (*unc-51-like kinase 4*) and *TRAK1* (*trafficking kinesin protein 1*), *MOB2* (*MOB kinase activator 2*), and *MAP3K4*. *ULK4* and *TRAK1* have been associated with feed conversion ratio in Duroc pigs [94]. *MOB2* has been reported to be associated with feed efficiency-related traits in Nellore cattle [95]. *MAP3K4* belongs to the family of *MAPK* genes, which are known to regulate the catalytic activity of kinases and phosphatases involved in the metabolism of phosphatidylinositols and inositol phosphates and cause serine phosphorylation of nuclear thyroid hormone receptor beta1; in Duroc pigs, gene and pathway analyses identified *MAPK* underlying residual feed intake and feed conversion ratio [94,96]. In our network analysis, *MAP3K4* was identified as sharing a protein domain with *BRSK2*, which plays essential roles in regulating several cellular processes, energy homeostasis, among the others. Potential functional similarities or interactions between the proteins encoded by these genes might be hypothesized. Feed efficiency expressed as residual feed intake has been reported to be negatively correlated with lean meat growth [97,98], thus selecting for feed efficiency may decrease backfat deposition and enhance body leanness.

**Table 1:** Candidate genes for ham weight loss at the end of curing; *Sus scrofa* chromosome (SSC), position of the gene (Mb), gene name, and functions are reported.

SSC	Position (Mb)	Gene	Name	Function	Reference
1	6.875–6.875	*AGPAT4*	1-acylglycerol-3-phosphate O-acyltransferases 4	Fat content and composition traits; fatty acid composition	[67]
1	6.919–6.919	*MAP3K4*	Mitogen-activated protein kinase 4	Feed efficiency traits	[94,96]
1	7.424–7.448	*IGF2R*	Insulin-like growth factor 2	Growth performance and carcass traits, muscle deposition, fat metabolism, meat production, and quality	[69]
1	15.228–15.231	*MTHFD1L*	Methylenetetrahydrofolate dehydrogenase (NADP+ dependent) 1 like	Ham weight loss at first salting	[10]
2	0.270–0.281	*PTDSS2*	Phosphatidylserine synthase 2	Backfat thickness, fat deposition	[68]
2	0.303–0.303	*HRAS*	HRas proto-onco, GTPase	Backfat thickness at slaughter, loin depth	[9,68]
2	0.466–0.466	*TALDO1*	Transaldolase 1	Lipid biosynthesis	[72]
2	0.545–0.545	*TSPAN4*	Tetraspanin 4	Cell development, activation, growth, and motility	[71]
2	0.633–0.633	*AP2A2*	Adaptor-related protein complex 2 subunit alpha 2	Linking lipids in the cell membrane	[93]
2	0.917–0.917	*BRSK2*	BR serine/threonine kinase 2	Backfat thickness	[68]
2	0.963–0.963	*MOB2*	MOB kinase activator 2	Feed efficiency	[95]
2	1.02–1.02	*DUSP8*	Dual specificity phosphatase 8	Ham weight loss at first salting	[10]
2	1.251–1.251	*TNNI2*	Troponin I2, fast skeletal type	Backfat thickness at slaughter; drip loss	[73,74,75]
2	1.264–1.289	*LSP1*	Lymphocyte-specific protein 1	Skeletal muscle development	[91]
2	1.251–1.251	*SYT8*	Synaptotagmin 8	Lipid metabolism	[76,77]
3	11.578–11.643	*GTF2IRD1*	GTF2I repeat domain-containing 1	Intramuscular fat content	[80]
3	11.674–11.786	*GTF2I*	General transcription factor IIi	Intramuscular fat content	[80]
5	63.717–63.717	*LPCAT3*	Lysophosphatidylcholine acyltransferase 3	Intramuscular and subcutaneous adipocytes	[70]
5	63.794–63.794	*ATN1*	Atrophin 1	Fat deposition in the early stages of development	[86]
5	63.860–63.860	*GNB3*	G protein subunit beta 3	Energy homeostasis and promotion of lipolysis	[87]
5	63.860–63.860	*CDCA3*	Cell Division Cycle-Associated 3	Body weight	[90]
6	6.697–6.697	*CMIP*	C-Maf-Inducing Protein	Lipid metabolism	[81]
8	3.245–3.317	*SORCS2*	Sortilin-related VPS10 domain-containing receptor 2	Backfat traits	[83]
8	128.628–128.795	*CCSER1*	Coiled-coil serine rich protein 1	Backfat thickness	[85]
8	131.075–131.075	*SPP1*	Secreted phosphoprotein 1	Body length, backfat thickness, loin muscle area	[89]
8	131.392–131.410	*SPARCL1*	SPARC-like 1	Calcium ion binding activity, proteolysis	[58]
12	55.166–55.166	*MYH8*	Myosin heavy chain 8	Calcium ion binding activity, proteolysis	[59]
13	25.730–25.730	*ULK4*	Unc-51-like kinase 4	Feed efficiency traits	[94]
13	25.864–25.930	*TRAK1*	Trafficking kinesin protein 1	Feed efficiency traits	[94]
15	34.488–34.886	*CSMD1*	CUB and sushi multiple domains 1	Cellular functions control, interaction with growth factors	[92]

## 4. Conclusions

Reaching an appropriate ham dehydration level after a long dry-curing process is of key importance to ensure high quality of the final product and to avoid economic losses, in particular for protected designation of origin productions. Locating genetic variants and detecting possible functional mechanisms that affect ham weight loss could facilitate the design of specific selection programs incorporating that information. Our genome-wide association study identified 221 loci located on almost all *Sus scrofa* chromosomes affecting ham weight loss at the end of dry-curing, each explaining a small percentage of the variance, thus confirming the polygenic nature of traits related to product dehydration such as ham weight loss at first salting. Functional annotations and genetic locations of significant windows revealed 32 windows as located within regulatory regions (enhancer, promoter, or open chromatin regions) and 94 windows located in intronic regions of specific genes. A candidate gene search led to the identification of 30 candidate genes encoding, among the others, receptors and enzymes associated with ham weight loss (*MTHFD1L* and *DUSP8*), proteolysis (*SPARCL1* and *MYH8*), drip loss (*TNNI2*), and fat metabolism (*AGPAT4, IGF2R, PTDSS2, HRAS, BRSK2, TNNI2, SYT8, GTF2I, GTF2IRD1, LPCAT3, ATN1, GNB3, CMIP, SORCS2, CCSER1, SPP1*). The results confirm the known relationship between dry-curing ham weight loss and fat content, suggesting that enhancing adipose tissue deposition might be key to improve ham curing aptitude.

## Data Availability

No data were deposited into official repositories. The data that support the findings of this study are available from Gorzagri (Fonzaso, Italy). Restrictions apply to the availability of these data, which were used under license for this study. Data are available from the authors upon reasonable request with the permission of Gorzagri.

## Supplementary Materials

The following are available online at https://www.mdpi.com/article/10.3390/ani14131983/s1, Table S1: Details of the SNPs included in the 221 windows explaining more than 0.3% of the variance; *Sus scrofa* chromosome (SSC), position of the SNP (BP), −log_10_(*p*-value), and percentage of variance explained by each SNP are reported. Table S2: Details of the 32 SNPs located in regulatory regions; *Sus scrofa* chromosome (SSC), position of the SNP (BP), and type of regulatory region (enhancer, promoter, open chromatin regions) are reported.
