# Supplementary material for: Genome-Wide Association Study for Weight Loss at the End of Dry-Curing of Hams Produced from Purebred Heavy Pigs
Source: Animals (Basel). 2024 Jul 5;14(13):1983. doi: 10.3390/ani14131983 (PMC11240668; doi:10.3390/ani14131983)
Supplement: Supplementary file 1 [file animals-14-01983-s001.zip › animals-3061950-supplementary.pdf]

Supplementary material

Table S1. Details of the SNPs included in the 221 windows explaining more than 0.3% of the variance; *Sus scrofa* chromosome (SSC), position of the SNP (BP), -log10(p-value) and percentage of variance explained by each SNP are reported.

SSC	Position (BP)	-log10(p-value)	% variance explained by the SNP
1	6875093	1.026	0.013
1	6919079	1.026	0.013
1	7423990	1.246	0.019
1	7448483	1.185	0.017
1	7487678	1.246	0.019
1	7511838	0.123	0.000
1	7623776	1.326	0.021
1	7659256	0.908	0.014
1	7676398	1.286	0.020
1	7692787	1.286	0.020
1	7717511	1.351	0.021
1	7734445	1.326	0.021
1	7734665	1.326	0.021
1	7765351	0.705	0.009
1	14859615	0.740	0.008
1	14946807	0.740	0.008
1	15024819	0.717	0.006
1	15028374	0.717	0.006
1	15045014	0.671	0.004
1	15046605	1.569	0.028
1	15099052	2.107	0.044
1	15111641	1.569	0.028
1	15131094	1.337	0.022
1	15228171	0.960	0.011
1	15231294	2.107	0.044
2	6982	3.487	0.061
2	84858	3.867	0.070
2	270027	4.532	0.100
2	281389	0.038	0.000
2	302556	3.892	0.071
2	466295	3.480	0.065
2	544535	3.375	0.063
2	563882	3.210	0.058
2	632707	3.480	0.065
2	830313	3.288	0.057

2	849587	3.288	0.057
2	917286	3.015	0.042
2	963130	3.015	0.042
2	980214	3.015	0.042
2	1001839	2.911	0.041
2	1021191	3.015	0.042
2	1048958	3.076	0.044
2	1067138	3.420	0.048
2	1086425	3.096	0.044
2	1129960	3.096	0.044
2	1226674	3.420	0.048
2	1250516	3.096	0.044
2	1264220	2.642	0.044
2	1289212	2.545	0.041
2	1396472	2.642	0.044
2	1416898	2.642	0.044
2	1422397	1.342	0.016
2	1432200	2.642	0.044
2	1457259	2.525	0.042
2	162084552	3.867	0.070
3	1951538	1.629	0.022
3	11484739	1.116	0.012
3	11519897	1.116	0.012
3	11578034	2.266	0.030
3	11614079	1.664	0.029
3	11643257	0.688	0.002
3	11673931	0.058	0.000
3	11728666	0.278	0.000
3	11745371	0.212	0.000
3	11754938	0.212	0.000
3	11786317	1.228	0.021
3	11820832	1.536	0.027
3	11829489	1.536	0.027
3	11843169	1.475	0.015
3	129202799	1.168	0.007
3	129220518	0.715	0.007
3	129273822	1.168	0.007
3	129351970	0.864	0.009
3	129411693	1.725	0.029
4	141084878	1.371	0.022
4	141168172	1.487	0.024
5	4049717	0.608	0.003

5	63702753	1.167	0.018
5	63717473	1.052	0.016
5	63794172	1.276	0.014
5	63860393	1.352	0.015
5	63917069	1.346	0.015
5	64711770	1.445	0.013
5	64817858	1.441	0.016
5	64861119	1.441	0.013
5	64876395	1.433	0.013
6	2048664	1.734	0.013
6	2122571	1.632	0.022
6	2148181	2.164	0.017
6	2195489	3.220	0.039
6	2223937	1.783	0.002
6	2303592	1.739	0.013
6	2308911	1.285	0.009
6	2333442	2.523	0.042
6	2370391	2.523	0.042
6	6648973	0.849	0.008
6	6697303	0.849	0.008
7	6375271	1.389	0.022
7	8185516	1.448	0.024
7	8294110	0.979	0.017
7	8316677	0.774	0.013
7	8326917	1.544	0.029
7	8517798	1.536	0.026
7	8563867	0.486	0.007
7	8566149	1.536	0.026
8	3244675	0.330	0.002
8	3245296	0.330	0.002
8	3278365	0.222	0.001
8	3305052	1.374	0.018
8	3317120	1.374	0.018
8	15732297	0.634	0.007
8	34849026	0.591	0.007
8	128628229	0.163	0.001
8	128652415	1.282	0.018
8	128681187	1.282	0.018
8	128724560	0.649	0.006
8	128761047	2.167	0.047
8	128777234	1.435	0.029
8	128794629	1.352	0.026

8	131053371	0.697	0.010
8	131075054	0.697	0.010
8	131095317	0.697	0.010
8	131233724	0.960	0.013
8	131247589	1.864	0.025
8	131392141	2.295	0.038
8	131398397	2.295	0.038
8	131410773	2.295	0.038
9	140046530	0.558	0.003
9	140099210	0.926	0.011
9	140127128	0.951	0.012
9	140160581	0.926	0.011
9	140242929	0.926	0.011
9	140253994	0.926	0.011
9	140273077	1.225	0.016
9	140284451	0.045	0.000
9	140368715	1.225	0.016
9	140376438	1.225	0.016
9	140769728	1.257	0.016
9	140900268	1.257	0.016
9	141062909	1.548	0.022
9	142246184	0.157	0.000
9	142307912	0.041	0.000
9	142322580	1.066	0.012
9	142338853	0.240	0.001
9	142356087	0.041	0.000
9	142369952	1.441	0.018
9	142472086	1.485	0.020
9	142491413	1.177	0.011
9	144064214	0.151	0.000
9	144086085	0.151	0.000
9	144138824	1.606	0.016
9	144161429	1.925	0.043
9	144185861	2.369	0.052
9	144200037	1.832	0.039
9	144285607	2.369	0.052
9	144355131	2.369	0.052
9	144377185	0.247	0.000
9	144408073	0.262	0.000
9	144445030	2.369	0.052
9	144458910	2.369	0.052
9	146850548	1.816	0.043

9	146924774	1.804	0.033
9	147037354	0.999	0.019
9	147037621	1.042	0.021
9	147130937	0.924	0.017
9	147145126	1.792	0.033
9	147232808	1.753	0.029
9	147421641	0.857	0.015
9	147505130	0.369	0.001
9	147628527	1.531	0.030
9	147742245	1.562	0.026
9	147755780	1.937	0.042
9	147926023	1.513	0.028
9	147971830	1.948	0.040
9	148130947	1.887	0.037
9	148280848	1.991	0.039
9	148284988	1.991	0.039
9	148296100	1.991	0.039
12	55166260	1.503	0.022
13	9452614	0.311	0.002
13	9482640	0.311	0.002
13	9497284	0.652	0.008
13	9510084	0.435	0.005
13	9518311	0.328	0.003
13	9554498	0.595	0.004
13	9565882	0.595	0.004
13	9580136	0.000	0.000
13	9591825	0.381	0.002
13	25730204	2.313	0.026
13	25765985	2.313	0.026
13	25864400	2.217	0.040
13	25929578	2.313	0.026
14	142022948	0.433	0.003
14	142033648	0.357	0.004
14	142051989	0.433	0.003
14	142055221	0.357	0.004
14	142067049	0.433	0.003
14	142076601	0.341	0.000
14	142089249	0.355	0.000
14	142163066	0.600	0.002
14	142174006	0.992	0.012
15	3894790	1.665	0.031
15	3962613	0.891	0.012

15	3969114	0.623	0.008
15	4002848	0.891	0.012
15	4036918	0.079	0.000
15	4061992	0.891	0.012
15	4109492	1.341	0.023
15	34488031	1.134	0.017
15	34548147	1.248	0.019
15	34613611	0.424	0.003
15	34635612	1.083	0.016
15	34672337	1.100	0.017
15	34697881	0.302	0.002
15	34736403	1.272	0.021
15	34802510	0.411	0.003
15	34820469	0.411	0.003
15	34834695	0.421	0.003
15	34850096	0.325	0.002
15	34868238	1.326	0.023
15	34885673	1.326	0.023

Table S2. Details of the 32 SNPs located in regulatory regions; *Sus scrofa* chromosome (SSC), position of the SNP (BP) and type of regulatory region (enhancer, promoter, open chromatin regions) are reported.

SSC	Position (BP)	Regulatory region
1	6875093	Enhancer
1	7423990	Open chromatin
1	7487678	Enhancer
1	7623776	Open chromatin
1	15024819	Enhancer
1	15099052	Open chromatin
2	84858	Open chromatin
2	281389	Enhancer
2	302556	Promoter
2	466295	Enhancer
2	544535	Open chromatin
2	563882	Open chromatin
2	632707	Open chromatin
2	1001839	Open chromatin
2	1021191	Open chromatin
2	1048958	Enhancer
2	1250516	Open chromatin
2	1264220	Open chromatin

2	1416898	Open chromatin
2	1432200	Enhancer
3	11643257	Enhancer
5	63794172	Enhancer
5	63860393	Open chromatin
6	2370391	Enhancer
7	8185516	Enhancer
7	8517798	Enhancer
7	8563867	Enhancer
8	3278365	Enhancer
8	3305052	Enhancer
8	131053371	Enhancer
8	131392141	Enhancer
13	25864400	Enhancer
